# Psychodermatology in Hungary: Awareness and practice patterns of dermatologists

**DOI:** 10.1002/ski2.419

**Published:** 2024-07-02

**Authors:** Gabriella Cápec, Mohammad Jafferany, Szergej Cápec, Sára Hoffmann, Miklós Sárdy

**Affiliations:** ^1^ Department of Pathological Physiology Faculty of Medicine and Dentistry Palacký University Olomouc Olomouc Czech Republic; ^2^ Department of Psychiatry and Behavioural Sciences Central Michigan University Saginaw Michigan USA; ^3^ Department of Dermatology, Venereology, and Dermatooncology Faculty of General Medicine Semmelweis University Budapest Hungary

## Abstract

**Background:**

Psychodermatology is an interdisciplinary branch of medicine between psychiatry /psychology and dermatology.

**Objectives:**

This study aimed to assess Hungarian dermatologists' awareness, practice patterns and attitudes towards psychodermatology.

**Methods:**

A questionnaire‐based survey was sent from October 2020 to June 2021 to 100 dermatologists (including residents in dermatology) in Hungary with a response rate of 51%. The participants were asked about understanding of the concept of psychodermatology and their training in this field, comfort in treatment, and interest in continuing medical education on dermatological disorders with psychological components.

**Results:**

49% of the respondents understood psychodermatology as a bidirectional interaction of the patient's mental and dermatological conditions, and 25.5% were comfortable in treating psychodermatological patients. The most common dermatological diagnoses with psychological/psychiatric involvement reported were psoriasis and atopic dermatitis. Atopic dermatitis, anxiety, delusional parasitosis, and depression were the most common diagnoses when dermatologists referred patients to psychiatrists. In total, 76.5% of participants could not name any resource (magazine, website, etc.) for patients and their families where information about psychodermatology is available, and 58.8% showed a definite interest in attending training on psychodermatology.

**Conclusion:**

The results of our study suggest that Hungarian dermatologists have a high awareness of the term “psychodermatology”, and only a quarter of practitioners feel adequately equipped and comfortable managing patients with psychosomatic aspects in their treatment. The relatively large gap between the number of patients with a psychodermatological component and referrals by dermatologists to mental health specialists indicates the potential importance of more intensive collaboration between dermatologists, psychiatrists and/or psychologists.

**What is already known?**

- There is a significant connection between psychological or psychiatric conditions and dermatological disorders, with up to 60% of dermatological patients having associated mental health problems.
- Psychodermatology is a discipline at the crossroads of dermatology and psychiatry that describes the relationship between mental health‐ and skin‐related conditions.
- Awareness and understanding of psychodermatology among dermatologists vary across different countries.

**What does this study add?**

- This is the first Hungarian survey to study current dermatologists' knowledge and understanding of psychodermatology.
- Hungarian dermatologists have a high awareness of the term "psychodermatology" in comparison to many other countries (USA, Turkey and the Balkans), but only a quarter of them are comfortable managing patients with psychosomatic aspects in their treatment.
- The most common dermatological diseases with a pronounced psychological component in Hungary are psoriasis, atopic dermatitis, and acne.

## 1 INTRODUCTION

Psychodermatology is an interdisciplinary part of medicine that studies the relationship between mental factors or diseases and dermatological manifestations and/or diseases.^1^, ^2^, ^3^, ^4^, ^5^, ^6^ The onset, persistence, and worsening of various skin conditions are significantly influenced by psychological factors, with increasing evidence pointing to a profound impact of skin diseases on the mental and emotional well‐being and quality of life of the patients and/or their caregivers.^7^, ^8^, ^9^, ^10^, ^11^, ^12^ Studies have demonstrated that about 30%–60% of dermatological patients have a psychological or psychiatric problem^1^, ^3^, ^5^, ^13^, ^14^, ^15^, ^16^; 85% of patients note a prominent role of psychological aspects in the course of their illness,^17^ and 98% note that their condition affects emotional and psychological well‐being.^6^, ^18^ Individuals with chronic skin conditions experience diminished quality of life and a diverse array of mental health challenges, spanning from low mood, anxiety, and depression to suicidal thoughts and psychoses.^9^, ^10^, ^12^, ^16^, ^19^, ^20^, ^21^, ^22^ Long‐term dermatologic diseases could also affect major life changing decisions such as marriage, divorce, having children, education and others.^6^ Psychodermatology has been actively developing relatively recently, but the relationship between "psycho" and the body, in particular the skin, was noticed even in the time of Hippocrates.^2^, ^23^, ^24^ The skin and the nervous system are closely related in embryogenesis, as both originate from the ectoderm and numerous neuroendocrine–immunological interactions connect these two organ systems.^1^, ^2^, ^3^, ^14^, ^25^, ^26^

Despite the common occurrence of various psychodermatological conditions, many patients are often underdiagnosed or undertreated for several reasons such as lack of availability of uniformly developed psychodermatology services, the need for training and education in this area among dermatologists and the patient's willingness to seek psychiatric help.^1^, ^3^, ^6^, ^27^, ^28^, ^29^

Over the past decade, many studies have been conducted in various countries worldwide such as Albania, Belarus, Canada, Egypt, India, Great Britain, Kazakhstan, Morocco, Turkey, Ukraine, United Arab Emirates, USA and more^1^, ^13^, ^27^, ^29^, ^30^, ^31^, ^32^, ^33^, ^34^, ^35^, ^36^, ^37^ to explore the current knowledge and the understanding of psychodermatology.

To the best of our current knowledge, this is the first study of its kind from Hungary.

## 2 MATERIALS AND METHODS

The study was carried out through an online survey. We contacted 100 dermatologists (including residents in dermatology) working in Hungary and sent them a link to a Google Form to complete the questionnaire. The study was conducted from October 2020 to June 2021.

The questionnaire used in the study was developed by one of the authors, validated for dermatologists in the USA^1^ and used in various similar studies in many countries—Ukraine, Belarus, Egypt, Kazakhstan, Albania, Turkey, United Arab Emirates, China and Iran.^13^, ^29^, ^30^, ^31^, ^32^, ^33^, ^34^, ^35^ In our survey the questionnaire was translated and adapted into Hungarian by two Hungarian native speaker co‐authors (GC and SH) and revised by the Hungarian native speaker senior author (MS).

The questionnaire consisted of 2 blocks. The first block included demographic data (age, gender, highest qualification, length and type of practice).

The second block included 11 questions related to psychodermatology. Two of them were open, where we asked participants to write down their ideas about what psychodermatology is and to name the sources of information (websites, books, etc.) on psychodermatology that may be useful for patients or their families.

The remaining 9 questions were in the form of multiple choice questions with the possibility of choosing one or more answers (depending on the question). The questions were related to participants' comfort level in treating patients with a psychological component, the number of such patients and the frequency of referral to psychiatrists/psychologists. They were also asked about the number and hours of training in psychodermatology and their interest and willingness to participate in activities to improve their skills in this area.

## 3 RESULTS

The questionnaire was sent to 100 participants, of which 51 were completed and returned back with a response rate of 51%. According to the results, almost half of the respondents (49%) understood psychodermatology as the interaction of the patient's mental and dermatological conditions in two directions, while 39% described psychodermatology as either the influence of mental status on dermatological status or vice versa (but not in both directions). This showed that most participants were aware of the term of psychodermatology and, probably, of the importance of psychological aspects in the pathogenesis and treatment of dermatological diseases. Only 12% of the respondents did not answer or demonstrated an inadequate understanding of the concept of psychodermatology.

Demographic data and general characteristics of survey respondents are shown in Table 1. Most of the participants who filled out the questionnaire were women (74.5%) and worked in the capital of Hungary, Budapest (82.4%). Among the survey respondents, the ratio of young doctors before specialization in dermatology was 49%, and 62.8% had less than 5 years of dermatology practice. A total of 70.6% of respondents had more than 30 patients per week, and only 3.9% had less than 10 patients in a week.

**TABLE 1:** Demographic and practice characteristics of respondents.

	Number of respondents	% of respondents
Age (years)		
<30 years	22	43.1
>60 years	3	5.9
31–40 years	16	31.3
41–50 years	6	11.8
51–60 years	4	7.8
Total	51	100.0
Gender		
Female	38	74.5
Male	13	25.5
Birth of place		
Hungary	47	92.1
Outside Hungary	4	7.8
Geographic location of practice		
Budapest	42	82.3
Budapest, Budapest agglomeration	4	7.8
Budapest, county seat/urban	1	2.0
County seat/urban	4	7.8
Title		
MD (before board exam)	25	49.0
MD (before board exam), PhD	2	3.9
MD (dermatologist)	7	13.7
MD (dermatologist), PhD	3	5.9
PhD	10	19.6
Professor	4	7.8
Length of practice (years)		
>10 years	15	29.4
0–5 years	32	62.7
6–10 years	4	7.8

Table 2 shows patterns in dermatological practice. A total of 88.2% of respondents had at least some experience with psychodermatology, including almost half of them (43.1%) having moderate or frequent experience. Moreover, 31.4% of respondents believed that less than 10% of patients have a psychodermatological component. At the same time, only a quarter of them (25.5%) felt comfortable in treating psychodermatological patients, while a third (33.3%) felt a different level of discomfort in treating such patients. According to Mann–Whitney *U* test, female dermatologists (FDerm) had lower comfort in treating psychodermatologic patients in comparison with male dermatologists (MDerm) (*p* < 0.001). It might be partially explained by the lower professional rank of FDerm participated in our research (*p* < 0.02 for the difference in the self‐reported professional grade with higher grade in MDerm).

**TABLE 2:** Practice patterns of dermatologists.

	Number of respondents	% of respondents
Experience with psychodermatology		
Frequent	4	7.8
Moderately common/somewhat	18	35.3
Occasional	23	45.1
None	6	11.8
Percentage of patients with psychocutaneous involvement		
<10%	16	31.4
>50%	1	2.0
10%–25%	19	37.3
26%–50%	15	29.4
Comfort level in treating of psychodermatologic patients		
Neutral	21	41.2
Somewhat comfortable	11	21.6
Somewhat uncomfortable	16	31.4
Very comfortable	2	3.9
Very uncomfortable	1	2.0

Table 3 shows the most frequent dermatologic disorders with a psychological component in the practices of survey respondents. Among the 6 diagnoses, psoriasis was the most common in our group of respondents.

**TABLE 3:** Common dermatologic disorders with psychiatric component.

Psychocutaneous disorder	0–5 patients/month	%	5–10 patients/month	%	>10 patients/month	%
Psoriasis	21	41.2	13	25.5	17	33.3
Atopic dermatitis	18	35.3	17	33.3	16	31.4
Acne	26	51	15	29.4	10	19.6
Vitiligo	42	82.4	6	11.8	3	5.9
Alopecia areata	42	82.4	7	13.7	2	3.9
Hyperhidrosis	42	82.4	5	9.8	4	7.8

This means that 33.3% of participants have more than 10 patients with psoriasis monthly who also have a psychological component in their disease. In our study, the second most frequent diagnosis with a psychodermatological component was atopic dermatitis (31.4% of participants had more than 10 such patients per month, and 33.3% of participants had 5–10 patients per month). Although many respondents noted that a large number of patients with various diagnoses had a mental component in their illness, more than 76% of survey respondents referred their patients to a psychologist or psychiatrist only once a year (54.9%) or even less often (15.7%). The most frequent reasons for which dermatologists referred their patients to a psychologist or psychiatrist (Table 4) were atopic dermatitis, anxiety, delusional parasitosis and depression (27.5%, 25.5%, 21.5% and 29.6%, respectively).

**TABLE 4:** Common diagnoses referred by dermatologists to psychiatrists.

Psychocutaneous disorder	Number of respondents	%
Atopic dermatitis	14	27.5
Anxiety (any kind of)	13	25.5
Delusion of parasitosis	11	21.6
Depression (any kind of)	10	19.6
Psoriasis	7	13.7
Artefact dermatitis	6	11.8
Prurigo	4	7.8
Alopecia (other types)	4	7.8
Skin tumours/melanoma	4	7.8
Trichotillomania	2	3.9
Body dysmorphic disorder, body image disorder	2	3.9
Anxiety secondary to skin disease	2	3.9
Stress‐related disorders	2	3.9
Miscellaneous	12	23.5

At the same time, 33 participants (64.7%) did not attend any trainings on the topic of psychodermatology.

In addition, more than ¾ (76.5%) could not name any resources (magazines, advocacy groups, websites, etc.) for patients and their families where information about psychodermatology was available.

The level of interest in training within this field was evident in the survey results, with only 2 participants (3.9%) expressing no inclination to participate in a dedicated event for physicians focusing on psychodermatology. Conversely, 58.8% responded affirmatively, while an additional 37.3% indicated a likelihood of attending. Most participants selected several topics that would interest them in such training courses. The greatest interest (Table 5) was shown concerning anxiety (secondary to the chronic skin disease) (70.6%), atopic dermatitis (62.8%), depression (60.8%), psoriasis (54.9%) and acne (54.9%).

**TABLE 5:** Interest in educational topics.

Topic	Respondents	%
Anxiety secondary to skin disease	36	70.6
Atopic dermatitis	32	62.7
Depression/Adjustment disorder due to skin disease	31	60.8
Psoriasis	28	54.9
Acne	28	54.9
Self‐injurious skin lesions	25	49.0
Alopecia areata	21	41.2
Trichotillomania	21	41.2
Delusion of parasitosis	20	39.2
Vitiligo	18	35.3
Body dysmorphic disorder	17	33.3
Hyperhidrosis	16	31.4
Diseases with chronic itching	1	2.0
Skin tumours	1	2.0
Anxiety due to skin tumours	1	2.0

## 4 DISCUSSION

As far as we are aware, this is the first study of its kind to evaluate dermatologists' knowledge, practical patterns, awareness, and interest in continuing medical education in the field of psychodermatology not only in Hungary but also in Central and Western Europe. All similar studies have been conducted in America, Asia, partly in Africa and Eastern Europe (Ukraine, Belarus).^1^, ^13^, ^16^, ^27^, ^29^, ^30^, ^31^, ^32^, ^33^, ^34^, ^35^, ^36^, ^37^

The response rate (51%) was higher than in North America (43% in the USA, 11% in Canada) but lower than in some Asian countries where studies were conducted (76.6% in Turkey, 65% in Iran, 60.7% in Kazakhstan).^1^, ^27^, ^29^, ^32^, ^35^

The most common cutaneous disorders with psychological components were psoriasis and atopic dermatitis (33.3% and 31.4% of survey respondents had more than 10 patients with these diagnoses/month, respectively). Acne was in the 3rd place. Among the dermatologists from the Washington State Dermatology Association, acne vulgaris was the most common disorder with a psychological component,^1^ followed by atopic dermatitis and psoriasis. In Turkey, Iran, Kazakhstan and Egypt, acne was also the most‐mentioned dermatologic disease with a psychiatric presentation followed by psoriasis, alopecia areata and other disorders.^29^, ^30^, ^32^, ^35^ The most common dermatoses with associated psychiatric involvement in many studies were similar in different countries. Comparing the results from Hungary, USA, Kazakhstan, Turkey, Iran, Egypt, Albania and Middle Eastern countries, acne seems to be among the top dermatoses in all the above mentioned countries.^1^, ^29^, ^30^, ^31^, ^32^, ^33^, ^35^ However, in the Hungarian study, this diagnosis is the 3rd most frequent (after psoriasis and atopic dermatitis), and in all other studies, it is at the first place. In addition to acne, the top 3 diagnoses include (albeit in a different order) mainly psoriasis, atopic dermatitis and alopecia.

The awareness of Hungarian dermatologists (49%) regarding the definition of the term "psychodermatology" is relatively high compared to other countries. This suggests that there is a growing interest in psychodermatology within the Hungarian medical community and may positively affect the quality of treatment of patients with dermatological problems. For comparison, only 16.2% and 18% of dermatologists in the USA and Eastern Europe, respectively, interpreted psychodermatology correctly, none of the respondents did so in Kazakhstan, 31.1% interpreted in India, 32%–33% in Turkey, 33% in the Middle East, 10% in the Balkans and 60.4% in Egypt.^1^, ^13^, ^29^, ^30^, ^31^, ^32^, ^33^, ^36^

The dermatologists' awareness on the degree of psychological component in skin disease patients in different countries is diverse and contrasting. In Hungary, around one‐third of dermatologists think that less than 10% of patients have a psychological component associated with skin disease, while a similar number believe it is in the range of 10%–25%, and another third think it is more than 25%. In the United States, only 5.8% of dermatologists think that over 25% of their patients have a psychodermatological component, while in Turkey, this number is 43.4%.^1^, ^32^ In India and China 46.7% and 57% of dermatologists, respectively, believe that less than 10% of their patients presented with psychocutaneous involvement.^34^, ^36^

It is interesting to note that while Hungarian dermatologists in this survey demonstrated a strong grasp of psychodermatology and encountered a significant number of patients with psychodermatologic aspects in their practice (in contrast to the USA, Canada, or Egypt), the rate of referrals to psychiatrists or psychologists remained relatively low.^1^, ^27^, ^30^ The general practice in Hungary today is that it is up to the subjective perception of the managing staff (primarily the dermatologist) to recognize the patients' psychological distress and its severity. Research has shown that the severity of psychological and somatic signs and symptoms often do not reflect each other proportionally.^38^ Our personal experience on this issue is that in case of emerging severe situations such as suicidal ideas, dermatologists always seek psychological/psychiatric help for their patients. However, they do not distribute questionnaires among their patients to obtain psychological and psychiatric history. In case of a patient's abnormal attitude or behaviour, they rather refer the patient to a psychologist or psychiatrist than to clarify the situation.

According to our survey, only 23.5% of dermatologists referred their patients to these specialists at least once a month, with 54.9% doing so more frequently than once a year, which is close to the frequency of referring patients to psychiatrists or psychologists in China and in Morocco (62.4% and 56.5%, respectively, once a year or rarer).^34^, ^37^ This may indicate limited access to psychological/psychiatric support for patients with dermatological problems or a need for more awareness among physicians about the importance of this aspect in treatment. In many other countries (e.g. USA, Turkey, India, and Iran), more dermatologists make such referrals. In the USA, for comparison, 68% of dermatologists refer their patients to psychiatry at least once a month; in Turkey, this number is 73.9%, in India, 53.4%, and in Iran, 61.5%.^1^, ^32^, ^35^, ^36^ It suggests that there may be opportunities to enhance collaboration between dermatologists and mental health professionals in Hungary to better address the psychological component of skin disease in patient care.

Hungarian dermatologists also note a relatively low level of comfort in treating psychodermatologic patients. This might suggest a dire need for enhancing dermatologists' proficiency in psychodermatology, developing their communication skills, and refining their approaches when dealing with such patients. Dermatologists from the USA and the Middle East (UAE and Saudi Arabia) feel the most comfortable with such patients.^1^, ^31^ Thus, it is possible that US dermatologists are comfortable with treating psychodermatological patients, even if they do not actively use the term psychodermatology. More than 80% of dermatologists in a survey study from the USA feel very comfortable or at least somewhat comfortable; however, in the Middle East, this number is around 70%.^1^, ^31^ In Hungary, the percentage of such dermatologists is only 25.5%. Moroccan, Indian, Chinese, Canadian, and Albanian dermatologists have slightly lower comfort levels (66.1%, 60%, 58.6%, 44.8%, and 34%, respectively) than the US and the Middle East, but higher than Hungary, Turkey and Egypt, where the vast majority of dermatologists describe their attitude as either "neutral" or somewhat and very uncomfortable (74.51%; 63.48% and 76.9%, respectively).^27^, ^30^, ^32^, ^33^, ^34^, ^36^, ^37^

The correlation analysis (Spearman's rank‐order correlation) revealed that with the improvement of qualification (from a doctor before board exam to a professor), the number of psychodermatologic patients in practice proportionally decreases (*ρ* = 0.34, *p* < 0.05), and the comfort in managing such patients increases (*ρ* = 0.28, *p* < 0.05). The relative decrease in the percentage of psychodermatologic patients in the practice of doctors with a higher rank may potentially be due to the admission of patients with more complex dermatological problems, with a more severe course or complications. This problem requires further research due to the small number of doctors surveyed in these groups and the contradictory results compared to the study in Kazakhstan.^29^ In Markabayeva's study, with increasing years of practice among dermatologists, the percentage of patients with a psychological component increased.^29^

The number of dermatologists who have never participated in any educational activity and/or training in psychodermatology (64.7%) is about the same or lower as in many other countries (63.1%, 64.6%, 78.1%, 79% and 88.4% in Iran, Egypt, China, Albania and Morocco, respectively) and higher than in North America (46.2% and 42% in Canada and USA, respectively) and India (42.2%).^1^, ^27^, ^29^, ^30^, ^33^, ^34^, ^35^, ^36^, ^37^ It is also important to note that in different countries the surveys were conducted in different years, which may have affected the results. Especially the Hungarian study was performed during the COVID‐19 pandemic time, which might have enhanced dermatologists' sensitivity for psychodermatology. A total of 58.8% of surveyed Hungarian dermatologists showed a definite interest in seeking training on psychodermatology. This roughly corresponds to the interest of dermatologists in Canada (55.1%), Eastern Europe (61.9%), and Turkey (57.1%–60%).^13^, ^27^, ^32^ At the same time, dermatologists in some other countries showed a higher interest in continuing education in this field (in Egypt–74.1%, in Albania–75.6%, in Middle East–78%, in China–84.5%, in India–86.7% and in Morocco–92.9%).^30^, ^31^, ^33^, ^34^, ^36^, ^37^ There has been a call from both the scientific and medical communities for the creation of complex psychodermatological care, but access to it is currently limited for most patients. In Hungary, the Psychodermatological Task Force Group of the Hungarian Dermatological Society was established in 2002, with the aim of raising professional awareness of the mental health problems, quality of life and the potential for a multidisciplinary approach to skin diseases.^39^ It is leading the education of young dermatologists in this field. Organized by European Society for Dermatology and Psychiatry postgradual programme including a Psychodermatology Diploma could be another a possible way of managing need of Hungarian dermatologists in more knowledge in psychodermatology.^6^, ^40^

From the point of view of medical practice, the work of dermatologists is often assisted by a clinical psychologist or a health psychologist, and if psychopharmacological prescription may be necessary, a psychiatrist is involved. Despite the growing awareness of psychodermatological approaches the structure of psychodermatological care in Hungary, the competences and roles of the participants are not yet defined from all aspects. There are still no joint dermatology/psychiatry clinics, but specialists cooperate with each other intensely.^41^

Almost every fourth participant who took part in our survey (23.5%) knew some Internet source on psychodermatology. Approximately the same number was reported in a similar study conducted in Egypt (25.5%) and a bit lower number in Indian survey (20%).^30^, ^36^ Participants from many other countries were significantly less knowledgeable (USA–10%, Middle East–10% and Turkey–4.5%–5.8%).^1^, ^31^, ^32^ While the surveys in Egypt and Hungary were performed in 2020, those in the USA, Turkey, and the Middle East were done between 2010 and 2013, potentially explaining the lower popularity of Internet resources in the earlier years. Regardless of the year and country of conducting the study the awareness of online resources in psychodermatology is quite low (4.5%–25.5%).^1^, ^30^, ^31^, ^32^, ^36^ This indicates a need for greater education and availability of information about Internet resources in psychodermatology for physicians and patients.

It is fascinating that the top diagnoses referred by dermatologists to psychiatrists in different countries demonstrated pronounced variation and did not always correlate with the disease prevalence. In Hungary, the top diagnoses were atopic dermatitis and anxiety, while in many other similar studies (USA, Turkey, Albania, Turkey and Middle East) these diagnoses were not among the top diagnoses at all.^1^, ^31^, ^32^, ^33^

In contrast, trichotillomania was among the most frequent diagnoses for which dermatologists referred their patients to psychiatrists in the US, Albania, China, India, Morocco, Ukraine, Belarus and the Middle East, while in the Hungarian study, this diagnosis did not make the top list at all.^1^, ^13^, ^31^, ^33^, ^34^, ^36^, ^37^

Depression due to skin diseases and delusion of parasitosis were almost the only diagnoses present in the top lists of dermatologists of various countries (in particular, the USA, Hungary, the Middle East, China, India, Eastern Europe and Turkey).^1^, ^13^, ^31^, ^32^, ^34^

## 5 LIMITATIONS

This study has some limitations due to the relatively small sample size. Most of the respondents who completed the survey were from Budapest, which does not necessarily accurately reflect the situation in whole Hungary. Therefore, it would be advisable to conduct further studies with the representation of all regions of Hungary, larger number of responses and inclusion of different specialist involved into the help to people with psychocutaneous diseases, such as psychiatrists and psychologists. Further, there may be some non‐respondent bias with the possibility that dermatologists who have some kind of interest in this field might have responded more frequently. Information bias could also be a limitation in our survey as the use of self‐reported free text answers to some questions might have arisen out of curiosity by some participants.

## 6 CONCLUSION

In this study, a questionnaire was distributed among Hungarian dermatologists to explore their understanding and practice in the field of psychodermatology. The survey results not only indicated the existence of a significant interest in this area among dermatologists but also revealed certain shortcomings and challenges they faced.

The results of our study showed that Hungarian dermatologists have a high awareness of the term "psychodermatology" in comparison to many other countries (USA, Turkey and the Balkans), but many of them had never participated in continuing medical education and/or formal training on psychodermatology. A quarter of participants felt adequately equipped and comfortable to manage patients with psychosomatic aspects in their treatment. The relatively large gap between the number of patients with a psychodermatological disorder and referrals by dermatologists to mental health professionals indicates the potential importance of more intensive collaboration between dermatologists and psychiatrists and/or psychologists. Closing this disparity could be partially achieved by integrating psychodermatology courses within the curricula for both dermatology and psychiatry residents. Additionally, organizing regular continuing medical education events focused on psychodermatology for residents and young dermatologists, alongside the establishment of liaison clinics between psychiatry and dermatology, and support of a multidisciplinary approach could greatly benefit patients with psychodermatological disorders and chronic skin conditions accompanied by psychological comorbidity.

## CONFLICT OF INTEREST STATEMENT

The authors declare no conflict of interest.

## AUTHOR CONTRIBUTIONS

**Gabriella Cápec**: Conceptualization (equal); data curation (equal); formal analysis (equal); funding acquisition (equal); investigation (equal); methodology (supporting); project administration (lead); writing – original draft (lead); writing – review & editing (equal). **Mohammad Jafferany**: Conceptualization (equal); methodology (lead); writing – review & editing (equal). **Szergej Cápec**: Conceptualization (supporting); data curation (equal); formal analysis (equal); funding acquisition (equal); investigation (supporting); writing – original draft (supporting); writing – review & editing (supporting). **Sára Hoffmann**: Data curation (equal); methodology (supporting); project administration (equal); writing – review & editing (supporting). **Miklós Sárdy**: Conceptualization (supporting); data curation (equal); investigation (lead); methodology (supporting); project administration (supporting); supervision (lead); writing – original draft (supporting); writing – review & editing (lead).

## ETHICS STATEMENT

Not applicable.

## Data Availability

The data underlying this article will be shared on reasonable request to the corresponding author.
